# The Conservation of Average Entropy Production Rate in a Model of Signal Transduction: Information Thermodynamics Based on the Fluctuation Theorem

**DOI:** 10.3390/e20040303

**Published:** 2018-04-21

**Authors:** Tatsuaki Tsuruyama

**Affiliations:** 1 Clinical Research Center for Medical Equipment Development, Kyoto University Hospital, Shogoin-kawahara-cho 54, Sakyo-ku, Kyoto 606-8057, Japan; tsuruyam@kuhp.kyoto-u.ac.jp; Tel.: +81-75-366-7694; Fax: +81-75-366-7660; 2 Department of Drug Discovery Medicine, Pathology Division, Graduate School of Medicine, Kyoto University, Yoshida-Konoe-cho, Sakyo-ku, Kyoto 606-8315, Japan

**Keywords:** signal transduction, fluctuation theorem, average entropy production rate

## Abstract

Cell signal transduction is a non-equilibrium process characterized by the reaction cascade. This study aims to quantify and compare signal transduction cascades using a model of signal transduction. The signal duration was found to be linked to step-by-step transition probability, which was determined using information theory. By applying the fluctuation theorem for reversible signal steps, the transition probability was described using the average entropy production rate. Specifically, when the signal event number during the cascade was maximized, the average entropy production rate was found to be conserved during the entire cascade. This approach provides a quantitative means of analyzing signal transduction and identifies an effective cascade for a signaling network.

## 1. Introduction

Cell signal transduction is a non-equilibrium process, which is characterized by the existence of signal transduction caused by a chemical reservoir of energetic metabolites, such as adenosine triphosphate (ATP). Systems biology has been developed to analyze the signal transduction network [1,2,3,4,5,6,7,8], including computational neural network studies [2,9,10]. Methodologies for analyzing gene expression associated with protein–protein interactions [11,12,13,14,15,16] and studies of cancer cells have been conducted [17,18]. Furthermore, cell–cell communication has been investigated from the point of view of signaling network [19,20,21]. Relationship between entropy and biological information has been extensively discussed on signal transduction network [22]. From statistical data of the correlation between expression of selected gene or proteins as a node in the network, a type of entropy has been considered in protein–protein interactions and in gene expression interactions [12,14,23]. “Single cell entropy” was recently introduced as the basis of the microstate of a single cell [24]. On the other hand, a significant amount of data on signal transduction has accumulated in the cell biology field [25,26,27,28,29,30,31,32,33,34,35,36,37,38,39], and quantitative analyses of signal transduction have been recently performed using an *Escherichia Coli* model [40,41,42,43]. Using an information theory of mutual information, Uda et al. have conducted quantitative analysis in the MAPK (mitogen-activated protein kinases) cascade [40,44]. Thus, various studies of cell signal cascade and network have been conducted.

We have reported analyses of the relationship between signal step occurrence/transition probability and step duration based on information theory [7,45,46]. In the current study, we focused on the entropy production rate in signal transduction [7,12,14,23,40,44,46,47]. The objective of our current study is to evaluate signal transduction efficiency from the perspectives of the average entropy production rate (AEPR) in individual steps in actual biochemical reaction kinetics [45,48]. Source coding theory for information transmission efficiency was discussed by Brillouin and Shannon [48,49], and Kullback and Leibler [50], who generalized Shannon’s entropy theory. For the purposes of our study, we introduce a model of signal transduction and define transitional probability of the individual steps, as well as step duration, in reference to the application of source coding theory and fluctuation theorem (FT) to signal transduction [40,41,42,43]. 

The signal events consist of a sequence of phosphorylation/dephosphorylation reactions of signaling molecules in a cell. We used suffixes *m* and *j* to represent the number of cascades and the step number. Here, a model of cascades is presented in Equation (1) [25,26,27,28,29,30,31,32,33,34,35,39]. In this model, the signaling molecule at step 1 of cascade *m*, denoted as *X_m_*_1_*,* extracellular ligand, induces the modification of the *X_m2_* receptor on the cell membrane, such as epidermal growth factor receptor (EGFR), into *X_m_*_2_*. Subsequently, *X_m_*_2_ activates *X_m_*_3_ in the same manner. In this way, the signaling molecule at the *j –* 1-th step of cascade *m*, denoted as *X_mj−_*_1_*,* induces the modification of *X_mj_* into X_mj_*. First, dephosphorylation of X_mj_* into *X_mj_* occurs spontaneously or via an enzymatic reaction catalyzed by the phosphatase (*Ph_mj−_*_1_; 1 ≤ *j* ≤ *n*), at the *j –* 1-th step of cascade *m,* and the pre-stimulation steady state is subsequently recovered. The signal step is described in our previous study is given as follows [7]:

(1) $$\begin{array}{cc} X_{m1}+X_{m2}+ATP\to X_{m1}+X_{m2}*+ADP & :k_{m1},1^{st}\mathrm{step}\\ Ph_{m1}+X_{m2}*\to Ph_{m1}\;+X_{m2}+Pi & :k_{-m1},-1^{st}\\ \ldots \ldots & \\ X_{mj-1}*+X_{mj}+ATP\to X_{mj-1}*+X_{mj}*+ADP & :k_{m,j-1},j-1-\mathrm{th}\;\\ Ph_{m,j-1}*+X_{mj}*\to Ph_{m,j-1}*\;+X_{mj}+Pi & :k_{m,-j-1},-j-1-\mathrm{th}\;\\ \ldots \ldots & \\ X_{m,n-1}*+X_{mn}+ATP\to X_{m,n-1}*+X_{mn}*+ADP & :k_{m,n-1},n-1-\mathrm{th}\\ Ph_{m,n-1}*+X_{mn}*\to Ph_{m,n-1}*\;+X_{mn}+Pi & :k_{m,-n-1},\;-n-1-\mathrm{th}\;\\ (1\leq m\leq M;1\leq j\leq n)\; \end{array}$$

The lowercase *m* represents the total number of the cascade. In (1), *k_m, j−_*_1_ and *k_m, -j−_*_1_ are the kinetic coefficients for the individual steps. ADP, and Pi represent adenosine diphosphate, and inorganic phosphate, respectively. Subsequently, we arrange the selected steps in chronological activation order. For instance, a cascade consisting of signaling molecule sequence is described as follows:

*X_m_*_1_*X_m_*_2_* *X_m_*_3_* *X_m_*_4_* *X_m_*_5_* *X_m_*_3_*X_m_*_2_*X_m_*_5_*X_m_*_4_ (2)

As the extracellular factor, or ligand, *X_m_*_1_, stimulates the cell system, phosphorylated receptor *X_m_*_2_* is tentatively increased. Subsequently, the increase in other molecules follows sequentially as shown in (2). The above sequence represents an order of the increase in concentration of signaling molecules. Here we consider *Ψ_m_*, the total number of distinct signal events that is described by a set of sequences (2). We define information *I,* derived as shown above, as the total number, *Ψ_m_,* of signal events in the cascade *m*,

(3) $$I=K\log\psi_{m}$$

Here, if we use the entropy unit, we take *K* = *k_B_*, Boltzmann’s constant, and *Ψ_m_* can be given as the total number of combinations of *X_mj_* and X_mj_* (1 ≤ *j* ≤ *n*). On the other hand, in information science, *K* is equivalent to log_2_*e*. Shannon defined the channel capacity as follows [49]:

(4) $$C=\max\underset{\tau \to \infty }{\lim}\frac{K\log\psi_{m}}{\tau }=\max\underset{\tau \to \infty }{\lim}\frac{I}{\tau }$$

**Figure 1:**
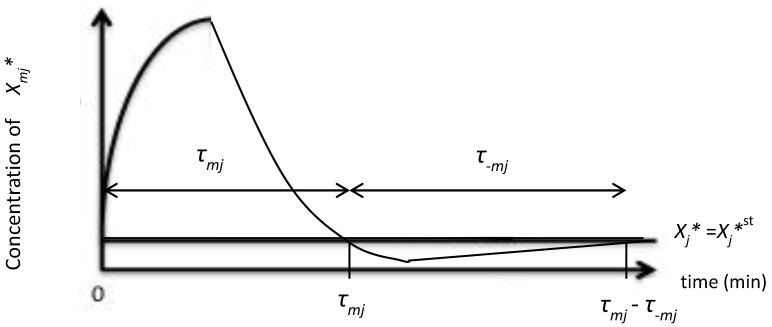
A common time course of the *j*-th step, showing fold changes during phosphorylation. The vertical axis denotes the concentration of signaling active molecule. The horizontal axis denotes the duration (min or time unit) of the *j-*th step. *τ_mj_* and *τ_−mj_* represent the duration of the *j*-th step and the *−j*-th step, respectively. The line X_j_* = X_j_*st denotes the concentration of X_j_* at the steady state.

As shown in Figure 1, we defined the duration, as forward *τ_mj_* and backward *τ_−mj_*. We assigned positive and negative value to *τ_mj_* and *τ_−mj_* to distinguish the direction of the step in the *m* cascade. *τ_mj_* represents the duration in which the active molecule X_mj_* tentatively increases in concentration, and *τ_−mj_* represents the duration in which the active molecule X_mj_* tentatively decreases in concentration. The individual step consists of both steps, and in the single *j*-th molecule the duration is represented by *τ_mj_* − *τ_−mj_*.

## 2. A Model of Signal Transduction

The occurrence probability, *p_mj_*, which represents the selection probability of *X_mj_* used in the *j*-th step in cascade *m* in the forward direction, takes the form of the *j*-th molecule. p_mj_*, which represents the selection probability of X_mj_*, used in the −*j*-th step for cascade *m* for backward direction in the cascade, as follows:

(5) $$\begin{array}{l} p_{mj}=X_{mj}/X\\ p_{mj}*=X_{mj}*/X \end{array}$$

with

(6) $$\sum_{j=1}^{n}\left(p_{mj}+p_{mj}*\right)=1$$

Here, *X* without suffix represents the total concentration of signaling molecules:

(7) $$X=\sum_{j=1}^{n}\left(X_{mj}+X_{mj}*\right)$$

The total concentration of non-phosphorylated signaling molecules is given by:

(8) $$\;\sum_{j=1}^{n}X_{mj}=X_{m}$$

and the total concentration of phosphorylated signaling molecules is given by:

(9) $$\;\sum_{j=1}^{n}X_{mj}*=X_{m}*$$

The entire duration, *τ_m_*, which signifies the sum of forward and backward cascades consisting of a set of signaling molecules, is determined by:

(10) $$\tau_{m}\;=\sum_{j=1}^{n}\left(X_{mj}\tau_{mj}–X_{mj}*\tau_{-mj}\right)$$

In Equations (5)–(9), we determined the entire duration using the probabilities *p_mj_* and p_mj_*. The entire duration is given here by:

(11) $$\tau_{m}\;=X\sum_{j=1}^{n}\left(p_{mj}\tau_{mj}–p_{mj}*\tau_{-mj}\right)$$

Subsequently, the total number of signal events, *Ψ_m_* is introduced as follows:

(12) $$\psi_{m}=X!/(\prod_{j=1}^{n}X_{mj}!\prod_{j=1}^{n}X_{mj}*!)\;$$

Using (5) and (6), Stirling’s approximation of (12), entropy *S_m_* is given as follows:

(13) $$S_{m}=\log\psi_{m}=-X\left(\sum_{j=1}^{n}p_{mj}\log p_{mj}+\sum_{j=1}^{n}p_{mj}*\log p_{mj}*\right)$$

To maximize *S_m_*, using non-determined parameters *α_m_,* and *β_m_,* in reference to the constraints established by Equations (6) and (11), we introduce a function *G* to apply Lagrange’s method for undetermined multipliers:

(14) $$\begin{array}{l} G\left(p_{m1},\;p_{m2},\;\cdots p_{mn};p_{m1}*,\;p_{m2}*,\;\cdots p_{mn}*;X\right)\\ =S_{m}-\alpha_{m}\sum_{j=1}^{n}\left(p_{mj}+p_{mj}*\right)-\beta_{m}\tau_{m}\\ =S_{m}-\alpha_{m}\sum_{j=1}^{n}\left(p_{mj}+p_{mj}*\right)-\beta_{m}X\sum_{j=1}^{n}\left(p_{mj}\tau_{mj}-p_{mj}*\tau_{-mj}\right) \end{array}$$

Then, we have

(15) $$\frac{\partial G}{\partial p_{mj}}=-X\left(\log p_{mj}-\beta_{m}\tau_{mj}\right)-\alpha_{m}-X$$

(16) $$\frac{\partial G}{\partial p_{mj}*}=-X\left(\log p_{mj}*+\beta_{m}\tau_{-mj}\right)-\alpha_{m}-X$$

(17) $$\frac{\partial G}{\partial X}=-\left(\sum_{j=1}^{n}p_{mj}\log p_{mj}+\sum_{j=1}^{n}p_{mj}*\log p_{mj}*\right)-\beta_{m}\left(\sum_{j=1}^{n}\tau_{mj}p_{mj}-\sum_{j=1}^{n}\tau_{-mj}p_{mj}*\right)$$

For maximization of *G*, setting the right sides of Equations (15)–(17) equal to zero gives

(18) $$-\log p_{mj}\;=\beta_{m}\tau_{mj}\;(\tau_{jm}>0)$$

(19) $$-\log p_{mj}*\;=-\beta_{m}\tau_{-mj}(\tau_{-mj}<0)$$

and

(20) $$\alpha_{m}=-X$$

Above, the two Equations (18) and (19) imply an important result that the coefficient *β_m_* is independent of the step number *j*.

## 3. Average Entropy Production Rate in a Signal Cascade and Fluctuation Theorem (FT)

We attempted to determine the parameter, *β_m_*, using thermodynamic parameters. *p_m_*(*j*|*j −* 1), the transitional probability of the *j*-th step given *j −* 1-th step, is defined. *v_m_*(*j*|*j −* 1), the transitional rate of the *j*-th step in a forward signaling direction, given *j −* 1-th step*,* is also defined. In addition, we define *p_m_* (−*j −* 1|−*j*) as the transitional probability of the –*j −* 1-th step given step −*j*-th step, and *v_m_* (*−j −* 1|*−j*) as the transitional rate of the –*j −* 1-th step in a backward signaling direction in a given cascade*,* given step −*j*-th step. The cell system is considered to stay at the detailed steady state, as follows:

(21) $$\begin{array}{l} p_{m}\left(j|j-1\right)v_{m}\left(j|j-1\right)\\ =p_{m}\left(-j-1|-j\right)v_{m}\left(-j-1|-j\right) \end{array}$$

Therefore, we have:

(22) $$\log\frac{p_{m}\left(-j-1|-j\right)}{p_{m}\left(j|j-1\right)}=\log\frac{v_{m}\left(j|j-1\right)}{v_{m}\left(-j-1|-j\right)}$$

From (1), using kinetic coefficients *k_mj_* and *k_−mj_*,

(23) $$\log\frac{p_{m}\left(-j-1|-j\right)}{p_{m}\left(j|j-1\right)}=\log\frac{k_{mj}X_{m,j-1}*X_{mj}ATP}{k_{-mj}Ph_{m,j-1}X_{mj}*}$$

Using (5),

(24) $$\log\frac{p_{m}\left(-j-1|-j\right)}{p_{m}\left(j|j-1\right)}=\log\frac{k_{mj}X_{m,j-1}*p_{mj}ATP}{k_{-mj}Ph_{m,j-1}p_{mj}*}$$

Dividing the both sides by *τ_mj_* − *τ_−__mj_* and taking the limit,

(25) $$\begin{array}{l} \underset{\tau_{mj}-\;\tau_{-mj}\to \infty }{\lim}\frac{1}{\tau_{mj}-\tau_{-mj}}\log\frac{p_{m}\left(-j-1|-j\right)}{p_{m}\left(j|j-1\right)}\\ =\underset{\tau_{mj}-\;\tau_{-mj}\to \infty }{\lim}\frac{1}{\tau_{mj}-\tau_{-mj}}\log\frac{p_{mj}}{p_{mj}*} \end{array}$$

Above, we set the parameters of the right side in (24) constant, except *p_mj_* and p_mj_*, because the parameters are supposed to be constants during the *j*-th step. Using Equations (18), (19), and (25), we have:

(26) $$\frac{1}{\tau_{mj}-\tau_{-mj}}\log\frac{p_{m}\left(j|j-1\right)}{p_{m}\left(-j-1|-j\right)}=-\beta_{m}$$

(27) $$\frac{1}{\left|\tau_{-mj}-\tau_{mj}\right|}\log\frac{p_{m}\left(-j-1|-j\right)}{p_{m}\left(j|j-1\right)}=\beta_{m}$$

Here, the AEPR $\langle \zeta_{mj}\rangle$ and $\langle \zeta_{-mj}\rangle$ during signal transduction is defined during *τ_mj_* − *τ*_−*mj*_ or |*τ*_−*mj*_ ‒ *τ_−__mj_*| for *m* cascade and reverse cascade –*m* using an arbitrary time parameter *t* :

(28) $$\langle \zeta_{mj}\rangle ≜\frac{1}{\tau_{mj}-\tau_{-mj}}\int_{0}^{\tau_{mj}-\tau_{-mj}}\zeta_{mj}(t_{mj})dt_{mj}$$

(29) $$\langle \zeta_{-mj}\rangle ≜\frac{1}{\left|\tau_{-mj}-\tau_{mj}\right|}\int_{0}^{\left|\tau_{-mj}-\tau_{mj}\right|}\zeta_{-mj}(t_{-mj})dt_{-mj}$$

From Equations (26)–(29), the FT gives

(30) $$\frac{1}{\tau_{mj}-\tau_{-mj}}\log\frac{p_{m}\left(j|j-1\right)}{p_{m}\left(-j-1|-j\right)}=\langle \zeta_{mj}\rangle$$

(31) $$\frac{1}{\left|\tau_{-mj}-\tau_{mj}\right|}\log\frac{p_{m}\left(-j-1|-j\right)}{p_{m}\left(j|j-1\right)}=\langle \zeta_{-mj}\rangle$$

Then, Equations (26), (27), (30), and (31) give

(32) $$\beta_{m}=-\langle \zeta_{mj}\rangle =\langle \zeta_{-mj}\rangle$$

where *β_m_* has dimension of entropy production rate and AEPRs are independent of the step number. AEPRs are redefined using (18) and (19) as follows:

(33) $$-\langle \zeta_{-mj}\rangle ≜\langle \zeta_{m}\rangle =-\frac{\log p_{mj}\;}{\tau_{mj}}$$

Equations (32) and (33) indicate conservation of entropy production rate during signal transduction. Accordingly, using *S_mj_* (= −log *p_mj_*) of the *j*-th step, we have:

(34) $$S_{mj}=\tau_{mj}\langle \zeta_{m}\rangle$$

Here, we obtained an important result that the channel capacity is given by AEPR. Accordingly, we obtained the following result from (18), (19), (32) and (33).

(35) $$-\log p_{mj}=\langle \zeta_{m}\rangle \tau_{mj}$$

(36) $$\log p_{mj}*=\langle \zeta_{m}\rangle \tau_{-mj}$$

In previous reports [7,45], we suggested a simple formulation between code occurrence probability *p_mj_* and duration *τ_mj_,* using an arbitrary parameter, ζ, which was independent of step numbers $-\log p_{mj}=\zeta \tau_{mj}$. In the current study, we developed the final proof and more detailed formulae of AEPR consistency based on source cording theory and FT as shown in Equations (35) and (36).

Therefore, from Equations (4), (11), (35) and (36), the channel capacity of the *m* cascade, *C_m_*, is given by:

(37) $$C_{m}=\max\underset{\tau_{m}\to \infty }{\lim}\frac{K\log\psi_{m}}{\tau_{m}}=K\langle \zeta_{m}\rangle$$

## 4. Conclusions

To maximize the signal event number at each signal step, that is to minimize code duration, we deduced a simple relational formula between the logarithm of the selection probability and the signal duration in Equations (18) and (19) [48]. Significantly, AEPR, <ζ*_m_>*, was independent of the step number and conserved during the whole cascade. In other words, AEPR is conserved in the model of signal transduction in which the signal transduction is performed in the most effective manner. 

Lapidus et al. [47] stated that having fewer fluctuations in rates leads to a more robust network and more energy efficiency. Our current conclusion is compatible with this. In the recent work from Sagawa and Ito [42,43], the entropy production rate is another important parameter for signal transduction and transmission that involves a feedback controller, Maxwell’s daemon. 

Further study will be required to prove which strategy of signal transduction the biological system will select [41,42]. In particular, the cell system may select a strategy to maximize signal event number during a given duration [45]—probably from a concern for metabolic efficiency, i.e., energetic cost in consumption of ATP—or it may select the strategy of maximizing accuracy in information transmission via a coding system with redundancy. Luo et al. actually measured the heat production in energy consumption in carbohydrate metabolism and were successful at measuring the consumption in normal and cancer cells; this can be applied to diagnosis of and therapy for cancer [18]. Another research has shown that sensory adaptation systems from a viewpoint of minimize cost; however, whether there are general thermodynamic principles governing cellular information processing remains unknown [51]. Considering that entropy production results from chemical substances from a chemical bath, such as ATP, channel capacity is a measurable quantity because information quantity can be discerned by measuring concentration changes of ATP. In a similar fashion, we can estimate the whole entropy production on the basis of the concentration change of ATP [46]. MAPK has been extensively studied and sufficient data have been reported [25,26,27,28,29,30,31,32,33,34,35,36,37,38,39]. Then, we have a planning of analysis based on the reported data as well as our own experimental data in future study. For the presented signal transduction, we developed a simple formula governing cellular signal transduction, the conservation of AEPR. In conclusion, this article’s information thermodynamic approach can provide a quantitative method of analyzing signal transduction.
